# Unilateral Sternalis With Double Slips: An Astounding Muscle, Often Unnoticed and Unknown

**DOI:** 10.7759/cureus.14185

**Published:** 2021-03-30

**Authors:** Sanjukta Sahoo, Suranjana Banik

**Affiliations:** 1 Anatomy, All India Institute of Medical Sciences, Bhubaneswar, Bhubaneswar, IND

**Keywords:** rectus sternalis, anatomical variation, breast augmentation, reconstruction, mammography, medical education

## Abstract

Discovery and variations of rectus sternalis muscle are occasionally seen in humans. However, during routine academic dissection of an adult male embalmed cadaver, a rare variant of the muscle was identified. The muscle had origin from the pectoral muscle and fascia and was inserted into external oblique aponeurosis along with the sixth rib and cartilage. It had double slips with the partial merging of the bellies. Knowledge regarding such unique muscle is important to anatomists for medical education as well as to surgeons during thoracic surgeries, in craniocaudal mammography where it can mimic breast mass and for using as muscle flap in the anterior chest wall, head and neck, and breast reconstructions.

## Introduction

Rectus sternalis is a name that we might have come across but not dealt with commonly. Often referred to as by multiple synonyms like Mystery Muscle, Sternalis Brottrum Episternalis, Presternalis, Rectus Thoracicus Superficialis, etc., this muscle mostly lies as a variant of the anterior chest wall, subcutaneously over pectoralis major [1,2]. It has been stated to be present in almost a large number of races and both sexes [3]. Ethnicity is reported to be in Asians, Blacks, and Caucasians. The highest incidence of 11.5% has been found in Asians and the maximum is seen in the Northern Chinese population comprising 18.2% and least in Taiwanese of 1%. Europeans are comprising 4.4% of incidence with Turkey alone having 9.3%. History says that in 1604 the muscle was first noticed by Carbolius, while in 1726, Du Puy gave a formal description of the muscle [4,5]. The 19th century saw work on this muscle by Albinus (Andrew) who referred to it as a rare example of nature’s playfulness - rarum naturae ludentis exemplum [6]. The first one to notice the prevalence of this muscle was Turner, who also noted its different arrangements [7]. As per earlier studies mostly the muscle has been found to be present superficially and perpendicular to the pectoralis major muscle and parallel to the sternum. It may be present unilaterally or bilaterally and can range from few short fibers to a well-developed muscle belly [4]. The identification of a muscle as the Sternalis is based on certain checklists like it should be located between superficial and deep fascia of the pectoral region. Its origin should be either from the upper sternum and the infraclavicular region or from pectoralis major or panniculus carnosus or rectus abdominis and variable insertion points such as the pectoral fascia, or lower ribs, or costal cartilages, or rectus abdominis muscle sheath or the abdominal external oblique muscle aponeurosis should be present [8]. There may be more than one origin and insertion too [4,8]. The blood and nerve supply are variable but most documented ones include either intercostal or medial pectoral nerve and intercostal artery and vein, respectively. This muscle has widespread clinical importance due to its location and its chances of getting misdiagnosed as benign and malignant anterior chest wall lesions and tumors [4].

In the present study, we report a unilateral rectus sternalis muscle with double slips to discuss its significance in clinical practice.

## Case presentation

**Figure 1:**
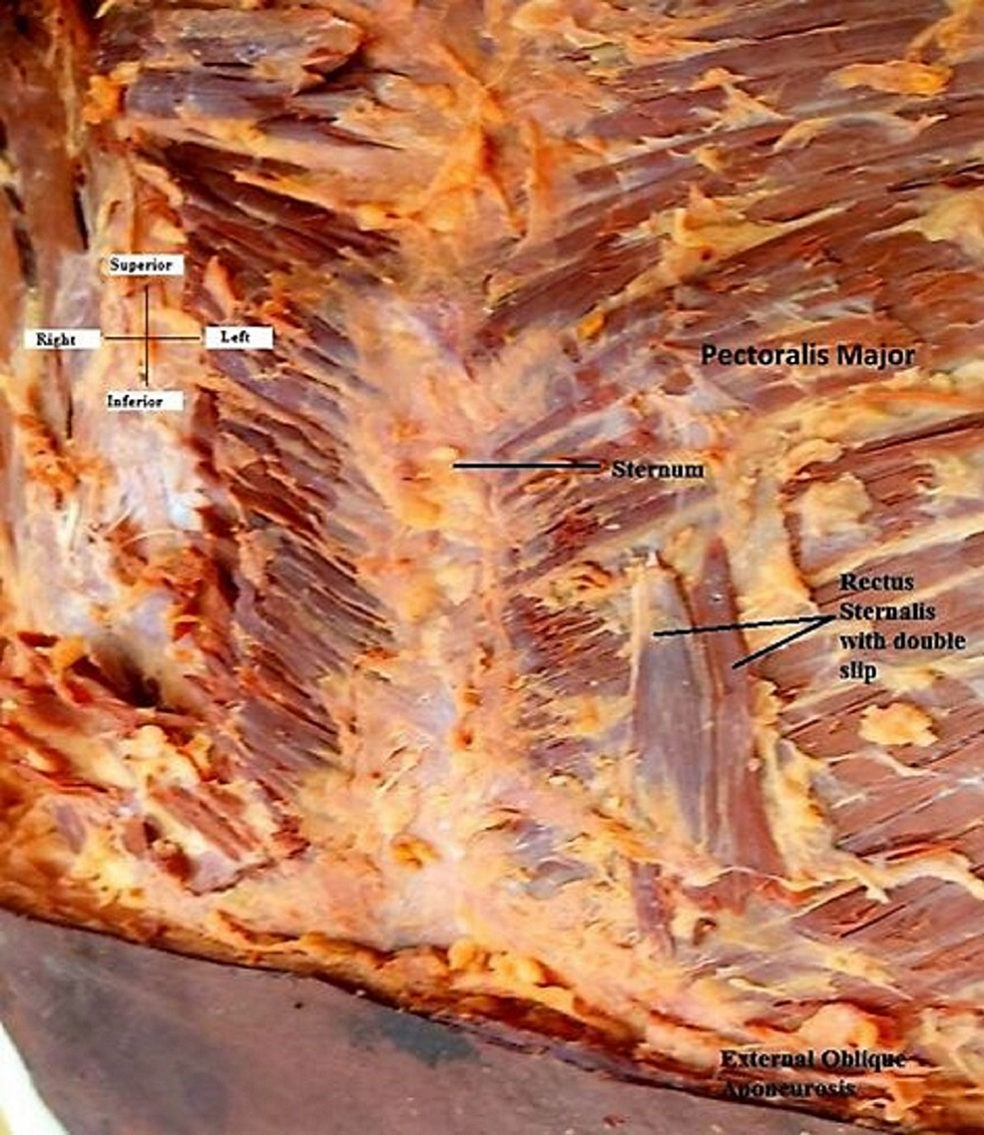
Anterior view of the specimen described in the present report Note the double slips of the rectus sternalis muscle and its origin from the pectoral fascia and upper segment of the pectoralis major.

The presence of the unilateral rectus sternalis muscle in a 69-year-old embalmed male cadaver on the left side was a serendipitous finding. It was found during routine undergraduate dissection curriculum at the Department of Anatomy, All India Institute of Medical Sciences, Bhubaneswar, India. The muscle qualified the checklists required to be called rectus sternalis, being present in between superficial and deep layers of pectoral fascia. The position of the muscle was parasternal, anterior to the pectoralis major muscle with its fibers directed parallel to the sternum and perpendicular to that of the pectoralis major (Figure 1). 

**Figure 2:**
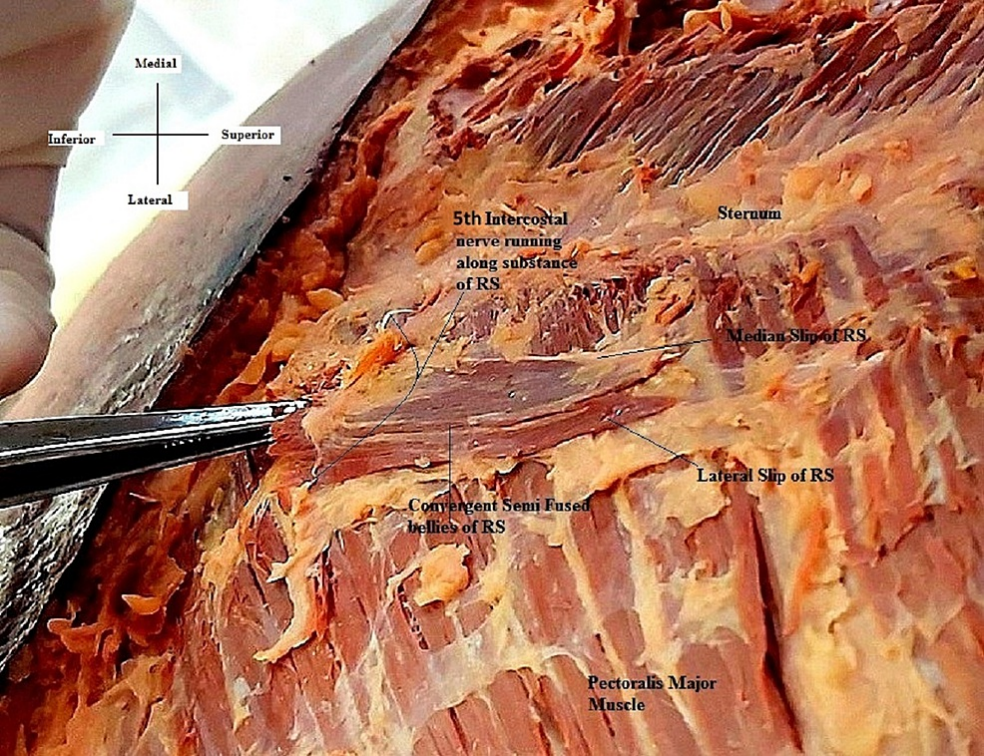
Close-up view of the rectus sternalis and its nerve supply Note the bellies of the muscle being pierced by the fifth intercostal nerve along with the arrangement of its fibers and slips.

In the upper end, it showed two well-separated slips and two bellies that were semi converged till the lower end. The fifth intercostal nerve was found to be supplying both, running transversely along the muscle bellies (Figure 2).

Both slips were equally developed, and the origin of both slips was from the upper segment of pectoralis major muscle and pectoral fascia, inferior to the clavicle and paramedian to the sternum at the level of fourth costal cartilage.

The insertion of both slips was at the sixth rib and sixth costal cartilage and external oblique aponeurosis. It measured 4 cm in length and 1.8 cm in width in the convergent part of the bellies. All measurements were done by digital vernier caliper with a precision of 0.01 mm.

## Discussion

The rectus sternalis is a quirky muscle having multiple variations in origin, insertions, nerve, and blood supply. Although common in quadruplets, in humans the incidence of muscle is occasional, with unilateral manifestation in 4.5% and bilateral in 1.7% [4].

As per the study by Getty, a rectus thoracis muscle in ruminants and horses that lies over the ventral chest wall and extends from the cephalad aspect of the rectus abdominis to the top of the sternum and which helps in the expansion of the chest and aggressive inhalation is described [9].

A similar muscle in higher primates has been noted and Osman Hill referred to this additional muscle as rectus sternalis, while Diogo and Wood, only in hominids, referred to the muscle as M sternalis as found in Homo and Hylobates syndactylus (the siamang) [10,11].

Embryologically, it is postulated to be a derivative of hypaxial myotomes/dermomyotomes. In case the muscle is originating from adjacent muscles, like pectoralis major or sternocleidomastoid, rectus abdominis, panniculus carnosus, etc., the blastema is considered a source of origin [4]. In the present study, the origin of the muscle was from the pectoralis major. Studies depict that in case of origin from the pectoral muscles, there remains a hidden defect in muscular patterning. The pre-pectoral mass in such defect migrates to form pectoralis major and minor and a separate muscle mass form the sternalis which due to mechanical disturbances, rotate atypically clockwise causing changes in muscle fibers direction [12,13].

As per the classification system of Ge et al. [14], the muscle in the present study belonged to category II due to having a double head/multiple heads with two bellies that eventually semi fuse. Again, as per Sonsek et al. [15], it belonged to a right bicipital convergent variant of Simple type with exception to its origin that is from pectoral fascia and muscle which makes it a novel variety.

Sternalis muscle does not follow the relationship between muscle ontogeny and innervation. External or internal thoracic nerve innervates the muscle in 55% of cases whereas 43% of cases are innervated by intercostal nerve and remaining by both [4]. Various authors suggested the microdissection technique as nerves are often destroyed in this region. In the present study, luck favored, and the innervation was preserved and found to be from the right fifth intercostal nerve.

The role of the rectus sternalis muscle is debated. It is found to play a role in lower chest elevation and thus can act as an accessory muscle of respiration. Alterations in electrocardiograms are seen in individuals with this muscle. Sternalis muscle is often mistaken as a tumor in the craniocaudal view of mammography too. It may cause breast or chest asymmetry or ipsilateral nipple-areola complex deviation. Sometimes other pectoralis major defects like Poland syndrome may coexist with it [4].

## Conclusions

Nature loves variation. It is these little changes that give us unique things to discover. Sternalis is one such little unique one. Though several studies have tried to jot down the peculiarities of the muscle, it seems to be resilient to regularity, and thus, we can still find new variations in different studies. Moreover, proper identification of the muscle is only possible when we have an idea about it. Thus, to enlighten the medicos, anatomists, surgeons, physicians about the muscle, its thorough evaluation is important. Addressing the less known will also help in medical education, and undergraduates and postgraduates of medical school can form a habit of noting down the anatomical variations that are beyond simple descriptions or morphology.
